# A 2:1 co-crystal of *p*-nitro­benzoic acid and *N*,*N*′-bis­(pyridin-3-ylmeth­yl)ethanedi­amide: crystal structure and Hirshfeld surface analysis

**DOI:** 10.1107/S2056989015024068

**Published:** 2016-01-01

**Authors:** Sabrina Syed, Siti Nadiah Abdul Halim, Mukesh M. Jotani, Edward R. T. Tiekink

**Affiliations:** a Department of Chemistry, University of Malaya, 50603 Kuala Lumpur, Malaysia; b Department of Physics, Bhavan’s Sheth R. A. College of Science, Ahmedabad, Gujarat 380001, India; c Centre for Crystalline Materials, Faculty of Science and Technology, Sunway University, 47500 Bandar Sunway, Selangor Darul Ehsan, Malaysia

**Keywords:** crystal structure, co-crystal, hydrogen bonding, carb­oxy­lic acid, thio­amide, Hirshfeld surface analysis

## Abstract

The title 2:1 co-crystal, 2C_7_H_5_NO_4_·C_14_H_14_N_4_O_2_, in which the complete di­amide mol­ecule is generated by crystallographic inversion symmetry, features a three-mol­ecule aggregate sustained by hydroxyl-O—H⋯N(pyrid­yl) hydrogen bonds. The *p*-nitro­benzoic acid mol­ecule is non-planar, exhibiting twists of both the carb­oxy­lic acid and nitro groups, which form dihedral angles of 10.16 (9) and 4.24 (4)°, respectively, with the benzene ring. The di­amide mol­ecule has a conformation approximating to a Z shape, with the pyridyl rings lying to either side of the central, almost planar di­amide residue (r.m.s. deviation of the eight atoms being 0.025 Å), and forming dihedral angles of 77.22 (6)° with it. In the crystal, three-mol­ecule aggregates are linked into a linear supra­molecular ladder sustained by amide-N—H⋯O(nitro) hydrogen bonds and orientated along [10-4]. The ladders are connected into a double layer *via* pyridyl- and benzene-C—H⋯O(amide) inter­actions, which, in turn, are connected into a three-dimensional architecture *via* π–π stacking inter­actions between pyridyl and benzene rings [inter-centroid distance = 3.6947 (8) Å]. An evaluation of the Hirshfeld surfaces confirm the importance of inter­molecular inter­actions involving oxygen atoms as well as the π–π inter­actions.

## Abstract 2

The components of the 2:1 co-crystal are linked by hy­droxy-O—H⋯N(pyrid­yl) hydrogen bonds into a three-mol­ecule aggregate having the shape of the letter Z. These are connected into a supra­molecular ladder by tight amide-N—H⋯O(nitro) hydrogen bonds.

## Chemical context   

Arguably, the most prominent motivation for the study of co-crystals relates to their potential applications in the pharmaceutical industry whereby co-crystals of active pharmaceutical ingredients (APIs) formed with generally regarded as safe (GRAS) co-crystal coformers might provide drugs with enhanced useful properties, *e.g*. stability, solubility, bioavailability, *etc*. (Aakeröy, 2015 ▸; Almarsson & Zaworotko, 2004 ▸). Further impetus for investigating co-crystals relates to ascertaining reliable supra­molecular synthons that might be exploited to direct crystal growth, or at least aggregates within crystals (Mukherjee, 2015 ▸; Tiekink, 2014 ▸). Co-crystals of *N*,*N′*-bis­(pyridin-3-ylmeth­yl)ethanedi­amide, see Scheme, figured prominently in early investigations of halogen bonding (*e.g*. Goroff *et al.*, 2005 ▸) and also has been co-crystallized with carb­oxy­lic acids (*e.g*. Nguyen *et al.*, 2001 ▸). As a continuation of recent work related to the study of co-crystal formation of pyridyl-containing mol­ecules with carb­oxy­lic acids (Arman *et al.*, 2013 ▸), the co-crystallization of *N*,*N′*-bis­(pyridin-3-ylmeth­yl)ethanedi­amide with *p*-nitro­benzoic acid was investigated, yielding the title 2:1 co-crystal. The results of this investigation are reported herein.

## Structural commentary   

**Figure 1:**
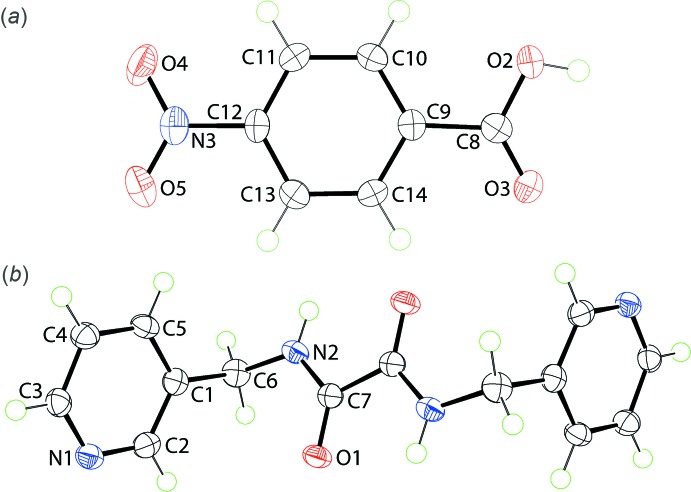
The mol­ecular structures comprising the 2:1 co-crystal in the title compound showing the atom-labelling scheme and displacement ellipsoids at the 70% probability level: (*a*) *p*-nitro­benzoic acid and (*b*) *N*,*N*′-bis­(pyridin-3-ylmeth­yl)ethanedi­amide; unlabelled atoms are related by the symmetry operation 2 − *x*, 1 − *y*, 2 − *z*.

The title co-crystal, Fig. 1 ▸, comprises a *p*-nitro­benzoic acid mol­ecule (hereafter, ‘acid’) in a general position, and a *N*,*N′*-bis­(pyridin-3-ylmeth­yl)ethanedi­amide mol­ecule (hereafter, ‘di­amide’) situated about a centre of inversion. This results in the 2:1 co-crystal stoichiometry.
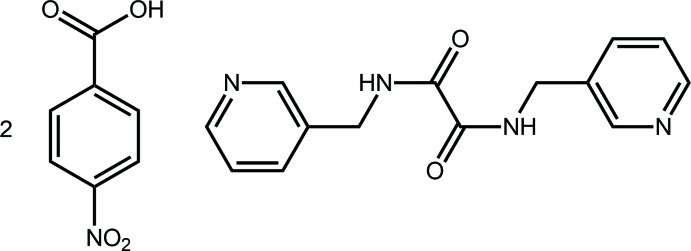


Twists are noted in the acid mol­ecule so that the dihedral angle between the benzene ring and the non-hydrogen atoms of the carb­oxy­lic acid group is 10.16 (9)°. The comparable angle involving the nitro group is 4.24 (4)°, consistent with a smaller twist. The substituents have a conrotatory disposition forming a dihedral angle of 13.50 (8)°. The crystal structure of the free acid was first reported almost fifty years ago (Sakore & Pant, 1966 ▸) and has been the subject of several subsequent investigations. The overall conformation for the acid in the title co-crystal matches the literature structures to a first approximation but it exhibits greater and smaller twists for the carb­oxy­lic acid and nitro groups, respectively, compared to those found in the two polymorphic forms of the free acid (*A*2/*a*: Tonogaki *et al.*, 1993 ▸; *P*2_1_/*m*: Bolte, 2009 ▸), Table 1 ▸.

The di­amide features an essentially flat central residue with the r.m.s. deviation for the eight non-hydrogen atoms (O1, N2, C6 and C7, and symmetry equivalents) being 0.025 Å. This planar arrangement allows for the formation of intra­molecular amide-N—H⋯O(amide) hydrogen bonds (Table 2 ▸). The pyridyl rings lie to either side of this plane and occupy positions approximately perpendicular to the plane, forming dihedral angles of 77.22 (6)°. Overall, the mol­ecule has the shape of a distorted letter Z. A number of co-crystals of the di­amide have been described and salient geometric parameters for these are collated in Table 3 ▸. All but one of these structures features a centrosymmetric di­amide mol­ecule. The range of dihedral angles between the central chromophore and the pendant pyridyl rings span the range 61.24 (5) to 84.6 (2)°. This conformational flexibility is reflected in the sole example of an organic salt of the di­amide where the two dihedral angles vary by approximately 18°, Table 3 ▸. The other feature of these structures worth highlighting are the relatively long central C—C bond lengths, their length often resulting in a *PLATON* (Spek, 2009 ▸) alert. In the structures included in Table 3 ▸, the central C—C bond lengths vary from 1.515 (3) to 1.550 (17) Å, *cf*. 1.530 (3) Å in the title co-crystal.

## Supra­molecular features   

**Figure 2:**
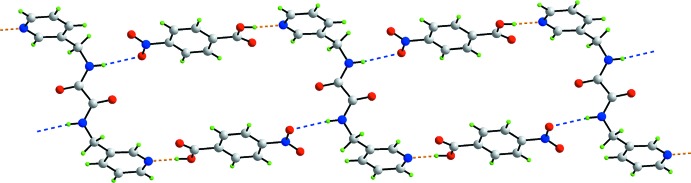
A view of the linear supra­molecular ladder in the mol­ecular packing of the title compound. The hydroxyl-O—H⋯N(pyrid­yl) and amide-N—H⋯O(nitro) hydrogen bonds are shown as orange and blue dashed lines, respectively.

**Figure 3:**
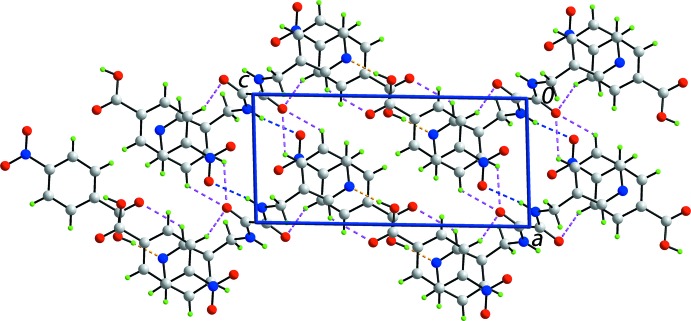
A view of the double layer in the title compound where the supra­molecular chains shown in Fig. 2 ▸ are connected by pyridyl- and benzene-C—H⋯O(amide) inter­actions, shown as pink dashed lines. The π–π inter­actions within the layers (see text) are not shown.

**Figure 4:**
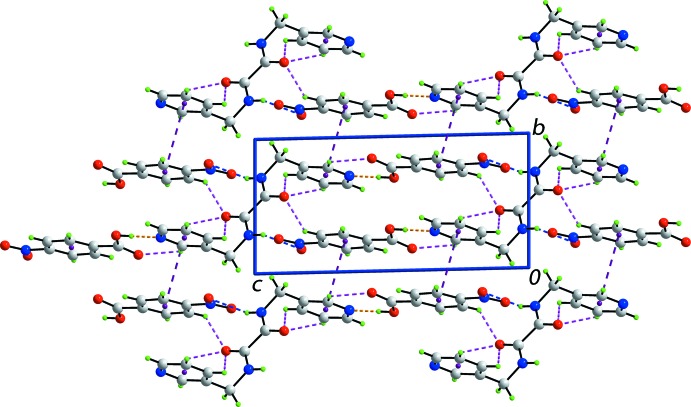
A view of the unit-cell contents of the title compound shown in projection down the *a* axis, whereby the supra­molecular layers, illustrated in Fig. 3 ▸, are linked by π–π inter­actions, shown as purple dashed lines, leading to a three-dimensional architecture.

In the packing, the anti­cipated (Shattock *et al.*, 2008 ▸) seven-membered {⋯HOCO⋯HCN} heterosynthon, featuring a strong hy­droxy-O—H⋯N(pyrid­yl) hydrogen bond and a complementary pyridyl-C—H⋯O(carbon­yl) inter­action, is observed, Table 2 ▸. By symmetry, each di­amide mol­ecule forms two such inter­actions, resulting in a centrosymmetric three mol­ecule aggregate. To a first approximation, the components of the aggregate are co-planar with the acid mol­ecules perpendicular to the di­amide mol­ecules, Fig. 2 ▸. This arrangement allows for the close approach of the nitro groups to the amide residues of translationally-related mol­ecules resulting in amide-N—H⋯O(nitro) hydrogen bonds, and the formation of supra­molecular ladders propagating along [10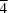]. This further results in the formation of centrosymmetric 36-membered {⋯HOC_5_NO⋯HNC_2_NC_3_N}_2_ supra­molecular rings, Fig. 2 ▸. The chains are linked into layers parallel to (010) by pyridyl-C—H⋯O(amide) inter­actions, and layers are connected into double layers by benzene-C—H⋯O(amide) inter­actions, as shown in Fig. 3 ▸. In this scheme, the amide-O1 atom accepts three close inter­molecular inter­actions. Further consolidation within the double layers is afforded by inter­actions of the type π–π, which occur between centrosymmetrically related pyridyl and benzene rings, *i.e. Cg*(pyrid­yl)⋯*Cg*(benzene)^i^ = 3.7214 (8) Å, with an angle of 4.69 (7)° between the rings; symmetry operation: (i) 2 − *x*, 1 − *y*, 1 − *z*. The connections between the layers to consolidate the three-dimensional architecture, Fig. 4 ▸, are also of the type π–π, and also occur between centrosymmetrically related pyridyl and benzene rings, *i.e. Cg*(pyrid­yl)⋯*Cg*(benzene)^ii^ = 3.6947 (8) Å with an angle of inclination = 4.69 (7)°; symmetry operation: (ii) 2 − *x*, 2 − *y*, 1 − *z*.

## Analysis of the Hirshfeld surfaces   

The packing of the title compound was also investigated by an analysis of the Hirshfeld surfaces (Spackman & Jayatilaka, 2009 ▸) with the aid of *CrystalExplorer* (Wolff *et al.*, 2012 ▸). The two-dimensional fingerprint plots (Rohl *et al.*, 2008 ▸) were calculated for the crystal as well as for the individual coformers, as were the electrostatic potentials using *TONTO* (Spackman *et al.*, 2008 ▸; Jayatilaka *et al.*, 2005 ▸), also with *CrystalExplorer*; the electrostatic potentials were mapped on the Hirshfeld surfaces using the STO–3G basis set at the level of Hartree–Fock theory over a range of ±0.075 au.

**Figure 5:**
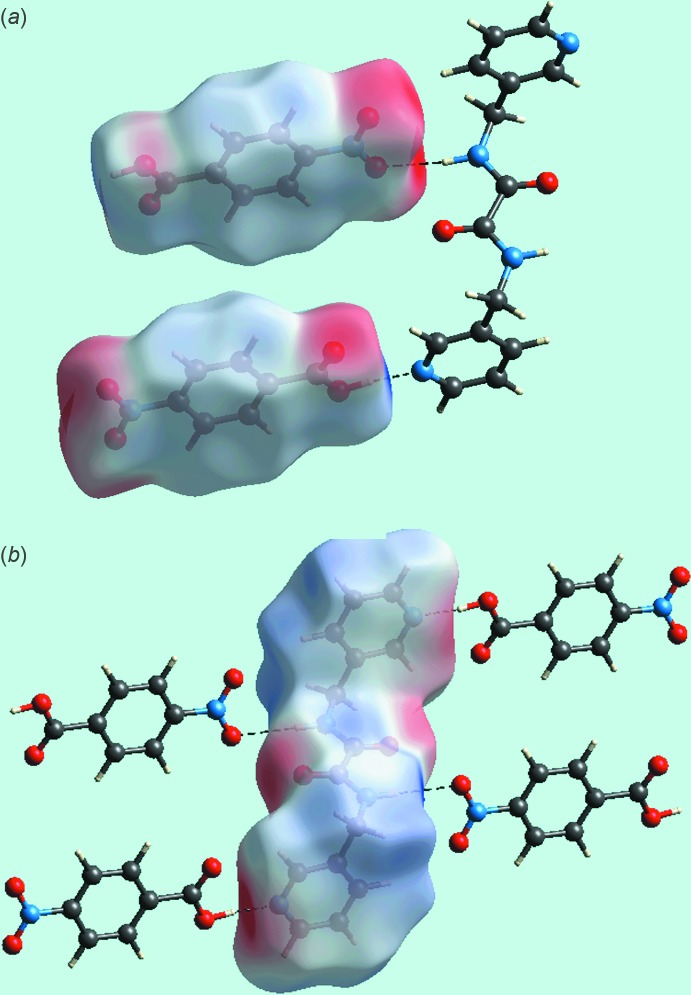
Views of the Hirshfeld surfaces mapped over the calculated electrostatic potential: (*a*) acid and (*b*) di­amide in the title compound.

**Figure 6:**
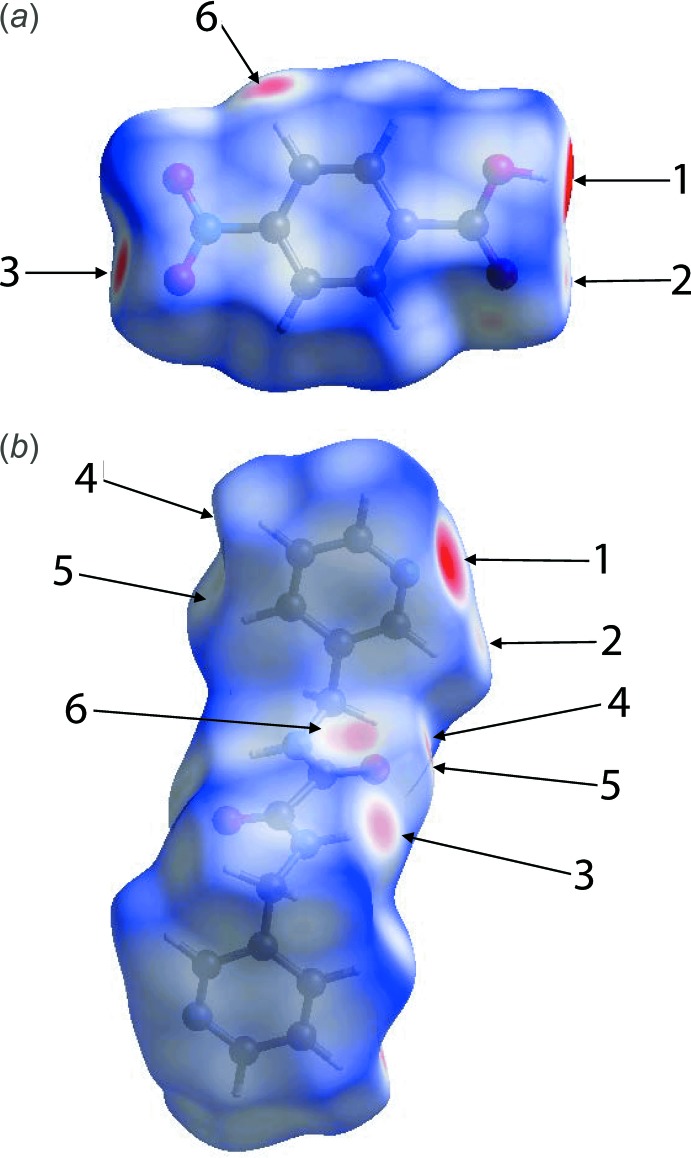
Views of the Hirshfeld surface mapped over *d*_norm_: (*a*) acid and (*b*) di­amide in the title compound.

**Figure 7:**
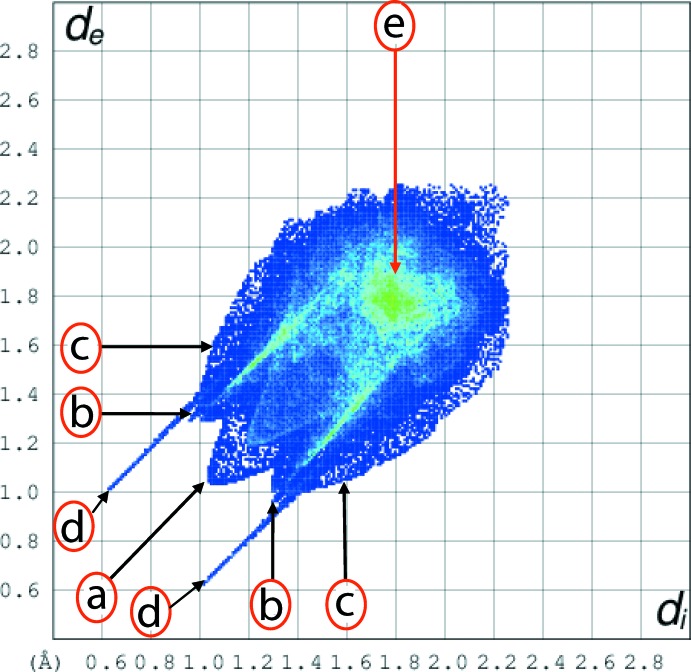
The two-dimensional fingerprint plot for the title 2:1 co-crystal showing contributions from different contacts: (a) H⋯H, (b) O⋯H/H⋯O, (c) C⋯H/H⋯C, (d) N⋯H/H⋯N and (e) C⋯C.

**Figure 8:**
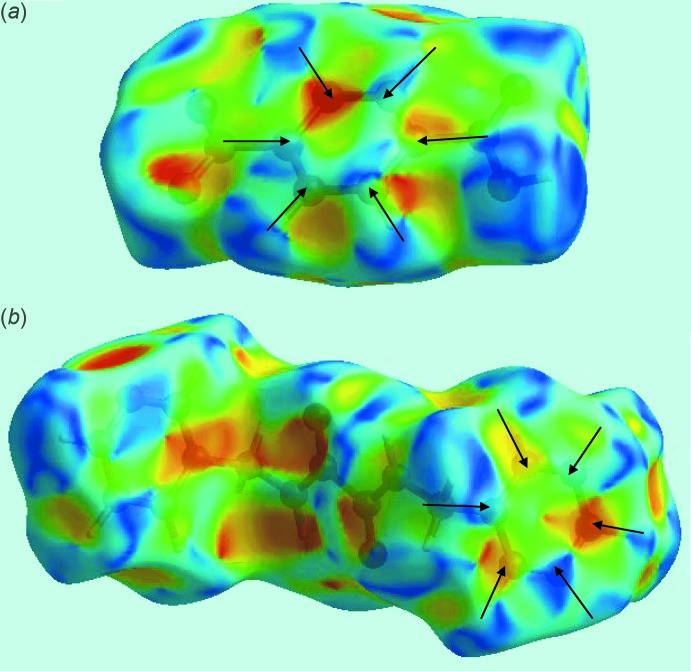
Hirshfeld surfaces mapped over the shape index for (*a*) the acid and (*b*) the di­amide, highlighting the regions involved in π–π stacking inter­actions.

**Figure 9:**
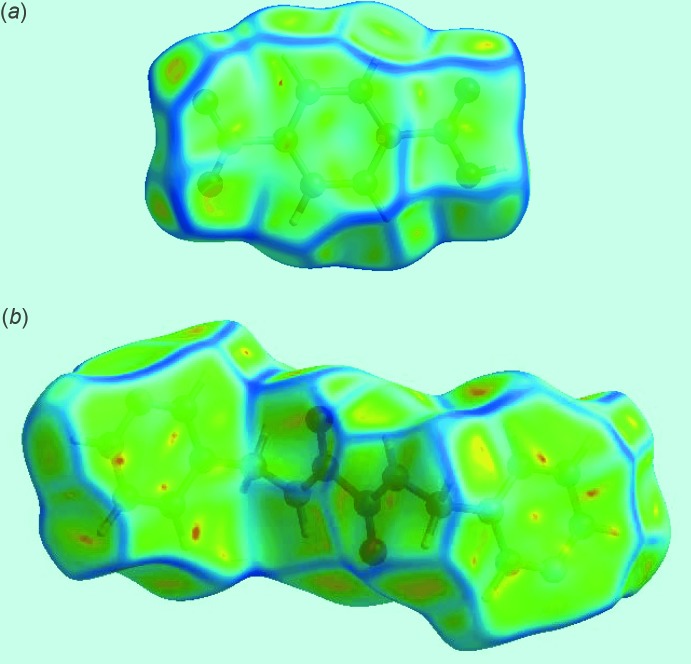
Hirshfeld surfaces mapped over curvedness for (*a*) the acid and (*b*) the di­amide, highlighting the regions involved in π–π stacking inter­actions.

The presence of strong hydroxyl-O—H⋯N(pyrid­yl) and amide-N—H⋯O(nitro) inter­actions between the pair of acid mol­ecules and a di­amide mol­ecule can be observed through their corresponding Hirshfeld surfaces mapped over the electrostatic potential, Fig. 5 ▸. From symmetry, each di­amide forms two pairs of inter­actions, visualized as bright-red spots on the Hirshfeld surface mapped over *d*_norm_ and labelled as 1 and 3, respectively, in Fig. 6 ▸. The full fingerprint plot for the co-crystal is shown in Fig. 7 ▸. The prominent spikes at *d_e_* + *d_i_* = 1.6 Å are due to N⋯H/H⋯N contacts. Thus, the long spike in the upper left region is due to the hydroxyl-O—H⋯N(pyrid­yl) inter­action and the similar long spike at the same *d_e_* + *d_i_* distance in the lower right region indicates the contribution of the amide-N—H⋯O(nitro) inter­action. The donor–acceptor contributions of these co-crystal constituents are highlighted with the label ‘d′ in the fingerprint plot, Fig. 7 ▸. The inter­molecular O⋯H and H⋯O contacts, which are prominent in the mol­ecular packing, Table 2 ▸ and Fig. 5 ▸, provide quite different contributions to the Hirshfeld surfaces of the acid and di­amide mol­ecules. While the O⋯H contacts have a large contribution, *i.e*. 32.3%, to the Hirshfeld surface of the acid, a smaller contribution, *i.e*. 8.3%, is provided by the di­amide; the reverse is true for for the O⋯H contacts, *i.e*. 8.5 and 30.4%, respectively. The overall fingerprint plot for the co-crystal when delineated into O⋯H/H⋯O contacts leads to the pair of spikes corresponding to donors and acceptors with a 37.1% contribution to surface (featured as ‘b’ in Fig. 7 ▸). The donors and acceptors corresponding to inter­molecular C—H⋯O inter­actions are seen as pale-red spots and are labelled as 2, 4, 5 and 6 in Fig. 6 ▸. The contribution from C⋯H/H⋯C contacts (10.4% of the Hirshfeld surface) results in a symmetrical pair of wings, see ‘c’ in Fig. 7 ▸. The C⋯C contacts assigned to π–π stacking inter­actions appear as a distinct triangle in the fingerprint plot, see ‘e’ in Fig. 7 ▸, at around *d_e_* = *d_i_* = 1.8 Å. The presence of these π–π stacking inter­actions is justified by the appearance of red and blue triangle pairs on the Hirshfeld surface mapped with shape index identified with arrows in the images of Fig. 8 ▸ and in the flat regions on the Hirshfeld surfaces mapped with curvedness in Fig. 9 ▸. The H⋯H contacts appear as the scattered points along with a single broad peak in the middle region of the fingerprint plot for each of the co-crystal constituents; the peak positions are at *d_e_* = *d_i_* = 1.2 and 1.0 Å, and the % contributions are 24.3% and 29.7% for acid and di­amide, respectively. Thus, the overall 28.6% contribution to the Hirshfeld surface of the co-crystal is just the superimposition of these individual fingerprint plots, and results in the peak marked with ‘a’ in Fig. 7 ▸. The relative contributions from various contacts to the Hirshfeld surfaces of acid, di­amide and the co-crystal are tabulated in Table 4 ▸.

A further analysis of Hirshfeld surfaces was conducted using a new descriptor, *i.e*. the enrichment ratio, ER (Jelsch *et al.*, 2014 ▸), Table 5 ▸. The ER relates to the propensity of chemical species to form specific inter­actions in the mol­ecular packing. The ER value of approximately 1.5 for the O⋯H/H⋯O contacts clearly provides evidence for the formation of O—H⋯N, N—H⋯O and C—H⋯O inter­actions. The high propensity of *N*-heterocycles, *e.g*. pyridyl, to form π–π stacking inter­actions with benzene is also evident from the high ER values corresponding to C⋯C contacts in the structure. On the other hand, the values of ER, *i.e*. < 0.6, reflects the low propensity for C⋯H/H⋯C contacts in the structure as the result of significant inter­actions involving O⋯H and N⋯H contacts. The enrichment ratios are closer to unity for the N⋯H/H⋯N contacts, an observation that is consistent with their relatively low contribution to the overall surface area. Finally, ER values close to but slightly less than unity for the H⋯H contacts are noted, in accord with expectation (Jelsch *et al.*, 2014 ▸). The ER values for other contacts are of low significance as they are derived from less important inter­actions with small contributions to the overall Hirshfeld surface.

## Database survey   

As mentioned in the *Chemical context*, the di­amide investigated herein has been the subject of several co-crystallization investigations, *i.e*. with co-formers capable of forming both halogen bonding and conventional hydrogen bonding inter­actions. Referring to the data in Table 3 ▸, three of the co-crystals having an iodide substituent in the co-former, feature N⋯I halogen bonding along with amide-N—H⋯O(amide) hydrogen bonding, the latter leading to amide ‘tapes’. The exceptional structure is found in the 1:1 co-crystal with *p*-C_6_F_4_I_2_ where I⋯O halogen bonding and amide-N—H⋯N(pyrid­yl) hydrogen bonding was observed (Hursthouse *et al.*, 2003 ▸). In co-crystals with carb­oxy­lic acids, hy­droxy-O—H⋯N(pyrid­yl) hydrogen bonding complementing amide-N—H⋯N(pyrid­yl) mediated tapes is normally observed. In the exceptional structure, *i.e*. of the 2:1 co-crystal with anthranilic acid, amide-N—H⋯O(carbon­yl) hydrogen bonding is seen along with hy­droxy-O—H⋯N(pyrid­yl) hydrogen bonding (Arman *et al.*, 2012 ▸). Finally, one salt has been reported with the 2,6-(NO_2_)_2_C_6_H_3_CO_2_^−^ anion (Arman *et al.*, 2013 ▸). Here, charge-assisted pyridinium-N—H⋯O(carboxyl­ate) and amide-N—H⋯O(carboxyl­ate) hydrogen bonding is found.

## Synthesis and crystallization   

The di­amide (0.5 mmol), prepared in accord with the literature procedure (Schauer *et al.*, 1997 ▸), in ethanol (5 ml) was added to a ethanol solution (5 ml) of 4-nitro­benzoic acid (Merck, 0.5 mmol). The mixture was stirred for 3 h at room temperature. After standing for a few minutes, a white precipitate formed which was filtered off by vacuum suction. The filtrate was then left at room temperature, yielding colourless blocks of the title compound after 2 weeks.

## Refinement   

Crystal data, data collection and structure refinement details are summarized in Table 6 ▸. The carbon-bound H-atoms were placed in calculated positions (C—H = 0.95–0.99 Å) and were included in the refinement in the riding model approximation, with *U*_iso_(H) set to 1.2*U*_equiv_(C). The oxygen- and nitro­gen-bound H-atoms were located in a difference Fourier map but were refined with distance restraints of O—H = 0.84±0.01 Å and N—H = 0.88±0.01 Å, and with *U*_iso_(H) set to 1.5*U*_eq_(O) and 1.2*U*_eq_(N).

## Supplementary Material

Crystal structure: contains datablock(s) I, global. DOI: 10.1107/S2056989015024068/hb7557sup1.cif

Structure factors: contains datablock(s) I. DOI: 10.1107/S2056989015024068/hb7557Isup2.hkl

Supporting information file. DOI: 10.1107/S2056989015024068/hb7557Isup3.cml

CCDC reference: 1442547

Additional supporting information:  crystallographic information; 3D view; checkCIF report

## Figures and Tables

**Table 1:** Dihedral angles (°) for *p*-nitro­benzoic acid in the title co-crystal and in poylmorphic forms reported in the literature

Structure	C_6_/CO_2_	C_6_/NO_2_	CO_2_/NO_2_	CSD refcode^*a*^	Ref.
*A*2/*a* form	2.37 (3)	14.82 (3)	17.34 (4)	NBZOAC04	Tonogaki *et al.* (1993 ▸)
*P*2_1_/*m* form	0.80 (10)	12.89 (9)	11.47 (12)	NBZOAC11	Bolte (2009 ▸)
Co-crystal	10.16 (9)	4.24 (4)	13.50 (8)	–	This work

Notes: (*a*) Groom & Allen (2014 ▸).

**Table 2:** Hydrogen-bond geometry (Å, °)

*D*—H⋯*A*	*D*—H	H⋯*A*	*D*⋯*A*	*D*—H⋯*A*
N2—H2*N*⋯O1^i^	0.88	2.35	2.7116 (16)	104
O2—H2*O*⋯N1	0.87 (1)	1.75 (1)	2.6114 (17)	175 (2)
N2—H2*N*⋯O5^ii^	0.88	2.36	3.2010 (17)	159
C2—H2⋯O3	0.95	2.56	3.2062 (19)	125
C4—H4⋯O1^iii^	0.95	2.37	3.0016 (18)	124
C5—H5⋯O1^iii^	0.95	2.54	3.0843 (18)	117
C11—H11⋯O1^iv^	0.95	2.45	3.2278 (18)	139

Symmetry codes: (i) 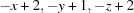; (ii) 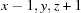; (iii) 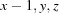; (iv) 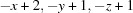.

**Table 3:** Selected geometric details (Å, °) for *N,*N*′*-bis­(pyridin-3-ylmeth­yl)ethanedi­amide mol­ecules, and halogen/hydrogen bonding in its organic co-crystals and a salt

Coformer	C_4_N_2_O_2_/pyrid­yl	C(=O)—C(=O)	Halogen/hydrogen bonding	Refcode^*a*^’	Ref.
*p*-C_6_F_4_I_2_	70.56 (7)	1.544 (4)	I⋯O; amide-N—H⋯N(pyrid­yl)	IPOSIP	Hursthouse *et al.* (2003 ▸)
IC CC CI	76.7 (2)	1.524 (10)	I⋯N; amide-N—H⋯O(amide)	WANNOP	Goroff *et al.* (2005 ▸)
IC CC CC CI	84.6 (2)	1.548 (11)	I⋯N; amide-N—H⋯O(amide)	WANPIL	Goroff *et al.* (2005 ▸)
{CC(I)=C(I)C CC(I)=C(I)C}_*n*_	80.6 (4)	1.550 (17)	I⋯N; amide-N—H⋯O(amide)	REWVUM	Jin *et al.* (2013 ▸)
[HO_2_CCH_2_N(H)C(=O)]_2_	64.4 (3)	1.532 (19)	hy­droxy-O—H⋯N(pyrid­yl); amide-N—H⋯O(amide)	CAJQAG	Nguyen *et al.* (2001 ▸)
[HO_2_CCH_2_N(H)]_2_C(=O)	81.47 (6)	1.515 (3)	hy­droxy-O—H⋯N(pyrid­yl); amide-N—H⋯O(amide)	CAJQEK	Nguyen *et al.* (2001 ▸)
[2-(CO_2_H)C_6_H_4_S]_2_	61.24 (5), 69.42 (6)^*b*^	1.534 (3)	hy­droxy-O—H⋯N(pyrid­yl); amide-N—H⋯O(amide)	KUZSOO	Arman *et al.* (2010 ▸)
2-NH_2_C_6_H_4_CO_2_H	74.95 (4)	1.543 (2)	hy­droxy-O—H⋯N(pyrid­yl); amide-N—H⋯O(carbon­yl)	DIDZAT	Arman *et al.* (2012 ▸)
2,6-(NO_2_)_2_C_6_H_3_CO_2_^−^	65.31 (4), 83.22 (5)^*c*^	1.541 (2)	pyridinium-N—H⋯O(carboxyl­ate); amide-N—H⋯O(carboxyl­ate)	TIPHAD	Arman *et al.* (2013 ▸)
4-NO_2_C_6_H_4_CO_2_H	77.22 (6)	1.530 (3)	hy­droxy-O—H⋯N(pyrid­yl)	–	This work

Notes: (*a*) Groom & Allen (2014 ▸). (*b*) The di­amide mol­ecule lacks a centre of inversion. (*c*) Salt: both pyridyl-N atoms are protonated in the non-symmetric dication.

**Table 4:** Percentage contribution of the different inter­molecular inter­actions to the Hirshfeld surfaces for the acid, di­amide and co-crystal

Inter­action	Acid	Di­amide	Co-crystal
H⋯H	24.3	29.7	28.6
O⋯H/H⋯O	40.8	38.7	37.1
C⋯H/H⋯C	10.5	7.6	10.4
N⋯H/H⋯N	4.5	8.2	4.4
C⋯C	6.5	7.0	7.2
C⋯N/N⋯C	4.4	4.6	5.0
C⋯O/O⋯C	6.5	3.1	5.4
N⋯O/O⋯N	0.4	0.4	0.4
O⋯O	2.1	0.7	1.5

**Table 5:** Enrichment ratios (ER) for the acid, di­amide and co-crystal

Inter­action	Acid	Di­amide	Co-crystal
H⋯H	0.89	0.92	0.96
O⋯H·H⋯O	1.55	1.56	1.48
C⋯H/H⋯C	0.59	0.46	0.54
N⋯H/H⋯N	0.94	1.09	0.82
C⋯C	3.26	2.20	2.90

**Table 6:** Experimental details

Crystal data	
Chemical formula	C_14_H_14_N_4_O_2_·2C_7_H_5_NO_4_
*M*_r_	604.53
Crystal system, space group	Triclinic, *P* 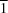
Temperature (K)	100
*a*, *b*, *c* (Å)	6.6981 (4), 6.9988 (4), 14.1770 (9)
α, β, γ (°)	91.070 (5), 92.131 (5), 96.602 (5)
*V* (Å^3^)	659.56 (7)
*Z*	1
Radiation type	Mo *K*α
μ (mm^−1^)	0.12
Crystal size (mm)	0.30 × 0.25 × 0.20

Data collection	
Diffractometer	Agilent SuperNova Dual diffractometer with Atlas detector
Absorption correction	Multi-scan (*CrysAlis PRO*; Agilent, 2014 ▸)
*T*_min_, *T*_max_	0.633, 1.000
No. of measured, independent and observed [*I* > 2σ(*I*)] reflections	5870, 2645, 2203
*R*_int_	0.021
(sin θ/λ)_max_ (Å^−1^)	0.628

Refinement	
*R*[*F* ^2^ > 2σ(*F* ^2^)], *wR*(*F* ^2^), *S*	0.040, 0.110, 1.05
No. of reflections	2645
No. of parameters	202
No. of restraints	2
Δρ_max_, Δρ_min_ (e Å^−3^)	0.37, −0.25

Computer programs: *CrysAlis PRO* (Agilent, 2014 ▸), *SHELXS97* (Sheldrick, 2008 ▸), *SHELXL2014*/7 (Sheldrick, 2015 ▸), *ORTEP-3 for Windows* (Farrugia, 2012 ▸), *QMol* (Gans & Shalloway, 2001 ▸), *DIAMOND* (Brandenburg, 2006 ▸) and *publCIF* (Westrip, 2010 ▸).

## supplementary crystallographic information

### Crystal data

**Table:**

C_14_H_14_N_4_O_2_·2C_7_H_5_NO_4_	*Z* = 1
*M**_r_* = 604.53	*F*(000) = 314
Triclinic, *P*1	*D*_x_ = 1.522 Mg m^−^^3^
*a* = 6.6981 (4) Å	Mo *K*α radiation, λ = 0.71073 Å
*b* = 6.9988 (4) Å	Cell parameters from 2741 reflections
*c* = 14.1770 (9) Å	θ = 4.0–28.8°
α = 91.070 (5)°	µ = 0.12 mm^−^^1^
β = 92.131 (5)°	*T* = 100 K
γ = 96.602 (5)°	Block, colourless
*V* = 659.56 (7) Å^3^	0.30 × 0.25 × 0.20 mm

### Data collection

**Table:**

Agilent SuperNova Dual diffractometer with Atlas detector	2645 independent reflections
Radiation source: SuperNova (Mo) X-ray Source	2203 reflections with *I* > 2σ(*I*)
Mirror monochromator	*R*_int_ = 0.021
Detector resolution: 10.4041 pixels mm^-1^	θ_max_ = 26.5°, θ_min_ = 2.9°
ω scan	*h* = −8→8
Absorption correction: multi-scan (*CrysAlis PRO*; Agilent, 2014)	*k* = −8→8
*T*_min_ = 0.633, *T*_max_ = 1.000	*l* = −17→16
5870 measured reflections	

### Refinement

**Table:**

Refinement on *F*^2^	2 restraints
Least-squares matrix: full	Hydrogen site location: mixed
*R*[*F*^2^ > 2σ(*F*^2^)] = 0.040	*w* = 1/[σ^2^(*F*_o_^2^) + (0.0513*P*)^2^ + 0.2196*P*] where *P* = (*F*_o_^2^ + 2*F*_c_^2^)/3
*wR*(*F*^2^) = 0.110	(Δ/σ)_max_ < 0.001
*S* = 1.05	Δρ_max_ = 0.37 e Å^−^^3^
2645 reflections	Δρ_min_ = −0.25 e Å^−^^3^
202 parameters	

### Special details

**Table:**

Geometry. All esds (except the esd in the dihedral angle between two l.s. planes) are estimated using the full covariance matrix. The cell esds are taken into account individually in the estimation of esds in distances, angles and torsion angles; correlations between esds in cell parameters are only used when they are defined by crystal symmetry. An approximate (isotropic) treatment of cell esds is used for estimating esds involving l.s. planes.

### Fractional atomic coordinates and isotropic or equivalent isotropic displacement parameters (Å^2^)

**Table:**

	*x*	*y*	*z*	*U*_iso_*/*U*_eq_	
O1	1.11011 (16)	0.56895 (15)	0.89399 (7)	0.0199 (3)	
N1	0.70817 (19)	0.71283 (17)	0.65579 (9)	0.0186 (3)	
N2	0.88281 (19)	0.71178 (17)	0.97708 (9)	0.0179 (3)	
H2N	0.8149	0.7109	1.0291	0.021*	
C1	0.7342 (2)	0.7881 (2)	0.82222 (11)	0.0172 (3)	
C2	0.8169 (2)	0.7782 (2)	0.73381 (11)	0.0183 (3)	
H2	0.9561	0.8193	0.7284	0.022*	
C3	0.5122 (2)	0.6562 (2)	0.66314 (11)	0.0187 (3)	
H3	0.4350	0.6101	0.6081	0.022*	
C4	0.4177 (2)	0.6621 (2)	0.74804 (11)	0.0194 (3)	
H4	0.2778	0.6218	0.7510	0.023*	
C5	0.5299 (2)	0.7276 (2)	0.82860 (11)	0.0178 (3)	
H5	0.4680	0.7313	0.8877	0.021*	
C6	0.8627 (2)	0.8617 (2)	0.90832 (11)	0.0204 (3)	
H6A	0.9980	0.9132	0.8883	0.024*	
H6B	0.8018	0.9684	0.9386	0.024*	
C7	1.0031 (2)	0.5762 (2)	0.96193 (10)	0.0155 (3)	
O2	0.87273 (16)	0.69092 (16)	0.49240 (8)	0.0215 (3)	
H2O	0.825 (3)	0.698 (3)	0.5481 (8)	0.032*	
O3	1.14230 (17)	0.83861 (17)	0.57361 (8)	0.0250 (3)	
O4	1.41048 (19)	0.74597 (18)	0.08648 (8)	0.0315 (3)	
O5	1.68626 (18)	0.82597 (18)	0.16957 (8)	0.0298 (3)	
N3	1.5028 (2)	0.78448 (18)	0.16264 (9)	0.0218 (3)	
C8	1.0608 (2)	0.7690 (2)	0.50090 (11)	0.0183 (3)	
C9	1.1723 (2)	0.7669 (2)	0.41044 (10)	0.0163 (3)	
C10	1.0748 (2)	0.7181 (2)	0.32349 (11)	0.0181 (3)	
H10	0.9339	0.6801	0.3201	0.022*	
C11	1.1823 (2)	0.7246 (2)	0.24197 (11)	0.0180 (3)	
H11	1.1166	0.6924	0.1823	0.022*	
C12	1.3883 (2)	0.7791 (2)	0.24931 (10)	0.0166 (3)	
C13	1.4902 (2)	0.8288 (2)	0.33481 (11)	0.0180 (3)	
H13	1.6310	0.8672	0.3379	0.022*	
C14	1.3796 (2)	0.8204 (2)	0.41546 (11)	0.0169 (3)	
H14	1.4458	0.8516	0.4751	0.020*	

### Atomic displacement parameters (Å^2^)

**Table:**

	*U*^11^	*U*^22^	*U*^33^	*U*^12^	*U*^13^	*U*^23^
O1	0.0189 (6)	0.0249 (6)	0.0158 (5)	0.0010 (4)	0.0052 (5)	0.0015 (4)
N1	0.0224 (7)	0.0171 (6)	0.0176 (7)	0.0061 (5)	0.0044 (6)	0.0030 (5)
N2	0.0194 (7)	0.0203 (6)	0.0138 (6)	0.0013 (5)	0.0032 (5)	−0.0007 (5)
C1	0.0206 (8)	0.0118 (6)	0.0196 (8)	0.0035 (6)	0.0012 (6)	0.0025 (6)
C2	0.0178 (8)	0.0149 (7)	0.0229 (8)	0.0031 (6)	0.0035 (6)	0.0045 (6)
C3	0.0211 (8)	0.0183 (7)	0.0174 (8)	0.0048 (6)	0.0000 (6)	0.0028 (6)
C4	0.0182 (8)	0.0191 (7)	0.0207 (8)	0.0010 (6)	0.0029 (6)	0.0032 (6)
C5	0.0208 (8)	0.0164 (7)	0.0169 (7)	0.0027 (6)	0.0053 (6)	0.0025 (6)
C6	0.0217 (8)	0.0166 (7)	0.0225 (8)	0.0003 (6)	0.0013 (7)	0.0009 (6)
C7	0.0133 (7)	0.0181 (7)	0.0138 (7)	−0.0025 (6)	−0.0017 (6)	−0.0040 (6)
O2	0.0197 (6)	0.0264 (6)	0.0185 (6)	0.0011 (5)	0.0052 (5)	0.0008 (5)
O3	0.0243 (6)	0.0333 (6)	0.0169 (6)	0.0014 (5)	0.0027 (5)	−0.0040 (5)
O4	0.0396 (8)	0.0406 (7)	0.0152 (6)	0.0078 (6)	0.0037 (5)	−0.0007 (5)
O5	0.0234 (7)	0.0353 (7)	0.0321 (7)	0.0051 (5)	0.0115 (5)	0.0037 (5)
N3	0.0273 (8)	0.0183 (6)	0.0215 (7)	0.0070 (6)	0.0066 (6)	0.0029 (5)
C8	0.0179 (8)	0.0158 (7)	0.0214 (8)	0.0031 (6)	0.0002 (6)	0.0027 (6)
C9	0.0190 (8)	0.0126 (7)	0.0177 (8)	0.0029 (6)	0.0025 (6)	0.0017 (6)
C10	0.0153 (7)	0.0162 (7)	0.0225 (8)	0.0010 (6)	0.0003 (6)	−0.0003 (6)
C11	0.0217 (8)	0.0163 (7)	0.0158 (7)	0.0024 (6)	−0.0024 (6)	−0.0004 (6)
C12	0.0212 (8)	0.0135 (7)	0.0161 (7)	0.0044 (6)	0.0052 (6)	0.0018 (6)
C13	0.0163 (8)	0.0161 (7)	0.0216 (8)	0.0022 (6)	0.0010 (6)	0.0011 (6)
C14	0.0190 (8)	0.0155 (7)	0.0158 (7)	0.0016 (6)	−0.0024 (6)	−0.0004 (6)

### Geometric parameters (Å, º)

**Table:**

O1—C7	1.2256 (17)	O2—C8	1.3145 (18)
N1—C3	1.335 (2)	O2—H2O	0.867 (9)
N1—C2	1.344 (2)	O3—C8	1.2150 (18)
N2—C7	1.3329 (19)	O4—N3	1.2338 (18)
N2—C6	1.4594 (19)	O5—N3	1.2294 (18)
N2—H2N	0.8800	N3—C12	1.4713 (19)
C1—C2	1.393 (2)	C8—C9	1.508 (2)
C1—C5	1.392 (2)	C9—C10	1.392 (2)
C1—C6	1.514 (2)	C9—C14	1.395 (2)
C2—H2	0.9500	C10—C11	1.383 (2)
C3—C4	1.383 (2)	C10—H10	0.9500
C3—H3	0.9500	C11—C12	1.388 (2)
C4—C5	1.384 (2)	C11—H11	0.9500
C4—H4	0.9500	C12—C13	1.387 (2)
C5—H5	0.9500	C13—C14	1.384 (2)
C6—H6A	0.9900	C13—H13	0.9500
C6—H6B	0.9900	C14—H14	0.9500
C7—C7^i^	1.530 (3)		
			
C3—N1—C2	118.66 (13)	N2—C7—C7^i^	113.82 (15)
C7—N2—C6	120.64 (13)	C8—O2—H2O	106.0 (13)
C7—N2—H2N	119.7	O5—N3—O4	123.07 (14)
C6—N2—H2N	119.7	O5—N3—C12	118.45 (13)
C2—C1—C5	117.70 (14)	O4—N3—C12	118.48 (13)
C2—C1—C6	121.03 (14)	O3—C8—O2	124.77 (14)
C5—C1—C6	121.28 (14)	O3—C8—C9	121.32 (13)
N1—C2—C1	122.83 (14)	O2—C8—C9	113.91 (13)
N1—C2—H2	118.6	C10—C9—C14	119.84 (14)
C1—C2—H2	118.6	C10—C9—C8	122.27 (13)
N1—C3—C4	122.32 (14)	C14—C9—C8	117.88 (13)
N1—C3—H3	118.8	C11—C10—C9	120.24 (14)
C4—C3—H3	118.8	C11—C10—H10	119.9
C3—C4—C5	119.07 (14)	C9—C10—H10	119.9
C3—C4—H4	120.5	C10—C11—C12	118.52 (14)
C5—C4—H4	120.5	C10—C11—H11	120.7
C4—C5—C1	119.42 (14)	C12—C11—H11	120.7
C4—C5—H5	120.3	C13—C12—C11	122.73 (14)
C1—C5—H5	120.3	C13—C12—N3	118.79 (13)
N2—C6—C1	112.31 (12)	C11—C12—N3	118.48 (13)
N2—C6—H6A	109.1	C14—C13—C12	117.71 (14)
C1—C6—H6A	109.1	C14—C13—H13	121.1
N2—C6—H6B	109.1	C12—C13—H13	121.1
C1—C6—H6B	109.1	C13—C14—C9	120.96 (14)
H6A—C6—H6B	107.9	C13—C14—H14	119.5
O1—C7—N2	124.82 (14)	C9—C14—H14	119.5
O1—C7—C7^i^	121.36 (17)		
			
C3—N1—C2—C1	0.5 (2)	O2—C8—C9—C14	−170.76 (12)
C5—C1—C2—N1	−0.2 (2)	C14—C9—C10—C11	−0.8 (2)
C6—C1—C2—N1	179.66 (13)	C8—C9—C10—C11	177.77 (13)
C2—N1—C3—C4	−0.1 (2)	C9—C10—C11—C12	0.5 (2)
N1—C3—C4—C5	−0.5 (2)	C10—C11—C12—C13	−0.5 (2)
C3—C4—C5—C1	0.8 (2)	C10—C11—C12—N3	179.50 (12)
C2—C1—C5—C4	−0.4 (2)	O5—N3—C12—C13	2.7 (2)
C6—C1—C5—C4	179.72 (13)	O4—N3—C12—C13	−177.78 (13)
C7—N2—C6—C1	75.28 (17)	O5—N3—C12—C11	−177.38 (13)
C2—C1—C6—N2	−113.64 (15)	O4—N3—C12—C11	2.2 (2)
C5—C1—C6—N2	66.21 (18)	C11—C12—C13—C14	0.8 (2)
C6—N2—C7—O1	3.1 (2)	N3—C12—C13—C14	−179.25 (12)
C6—N2—C7—C7^i^	−176.55 (14)	C12—C13—C14—C9	−1.0 (2)
O3—C8—C9—C10	−168.99 (14)	C10—C9—C14—C13	1.0 (2)
O2—C8—C9—C10	10.7 (2)	C8—C9—C14—C13	−177.57 (13)
O3—C8—C9—C14	9.6 (2)		

Symmetry code: (i) −*x*+2, −*y*+1, −*z*+2.

### Hydrogen-bond geometry (Å, º)

**Table:**

*D*—H···*A*	*D*—H	H···*A*	*D*···*A*	*D*—H···*A*
N2—H2*N*···O1^i^	0.88	2.35	2.7116 (16)	104
O2—H2*O*···N1	0.87 (1)	1.75 (1)	2.6114 (17)	175 (2)
N2—H2*N*···O5^ii^	0.88	2.36	3.2010 (17)	159
C2—H2···O3	0.95	2.56	3.2062 (19)	125
C4—H4···O1^iii^	0.95	2.37	3.0016 (18)	124
C5—H5···O1^iii^	0.95	2.54	3.0843 (18)	117
C11—H11···O1^iv^	0.95	2.45	3.2278 (18)	139

Symmetry codes: (i) −*x*+2, −*y*+1, −*z*+2; (ii) *x*−1, *y*, *z*+1; (iii) *x*−1, *y*, *z*; (iv) −*x*+2, −*y*+1, −*z*+1.
